# Feasibility of the breastfeeding peer support application—perspectives of peer supporters and breastfeeding mothers

**DOI:** 10.1186/s13006-025-00785-7

**Published:** 2025-12-29

**Authors:** Riikka Ikonen, Hannakaisa Niela-Vilen

**Affiliations:** 1 Faculty of Social Sciences, Tampere University, Tampere, Finland; 2 Faculty of Medicine, Department of Nursing Science, University of Turku, Turku, Finland

**Keywords:** Application, Breastfeeding, Breastfeeding mother, Peer support, Peer supporter

## Abstract

**Background:**

Online breastfeeding peer support, which provides information, advice, emotional support, reassurance, and a sense of shared experience, is increasingly delivered through mobile applications as a relatively new form of digital peer support. This study examined the feasibility of a breastfeeding peer support application from the viewpoints of peer supporters and breastfeeding mothers.

**Methods:**

A feasibility study with both qualitative and quantitative data, combining the perspectives of peer supporters and breastfeeding mothers, was conducted in Finland in 2022. The qualitative data were collected from breastfeeding peer supporters (*n* = 8) via focus groups and analyzed via content analysis. The quantitative data from breastfeeding mothers (*n* = 81) were collected via a structured survey embedded in the Breastfeeding Peer Support Application and analyzed with descriptive statistics.

**Results:**

Four main categories describing the perspective of eight experienced peer supporters were created. First, controlling the burden of peer support was possible, as peer supporters were able to regulate their availability in the App. Second, peer supporters had a strong sense of duty, and some of the features of the App helped them avoid overusing their resources, but others did not. Thus, the peer supporters had to balance the sense of duty and the features of the App. Third, peer support contacts with breastfeeding mothers were mostly rewarding, but a few suddenly breaking and disappearing contacts caused frustration and insecurity. Both short and easy-to-solve contacts and long-term contacts were rewarding. The fourth main category illustrates that real-time support via the App is present and responds well to the increasing need for online support. On the basis of the survey completed by breastfeeding mothers (*n* = 81), they were satisfied with the App; it was ranked high in acceptability, appropriateness and usability regardless of mothers’ age, education or number of children.

**Conclusions:**

The breastfeeding peer support application was shown to be a feasible tool from the perspectives of both trained peer supporters and breastfeeding mothers and was easily integrated into their daily lives. Most mothers found effective solutions to their breastfeeding challenges. Overall, the App was regarded as a highly promising tool for supporting breastfeeding.

**Trial registration:**

N/A.

## Background

For breastfeeding mothers, multifaceted support is essential to achieve their own breastfeeding goals. Breastfeeding peer support, defined as informational and emotional support provided by persons with both their own experience and training [1, 2], supplements the counseling provided by health care professionals [3]. In addition, it provides unique viewpoints of practical knowledge and skills [4], lifestyle compatibility [5], parenting skills [6], and a sense of community [7]. Until recently, peer support has been organized as group meetings [8] or one-to-one contact [9]. Currently, especially in the postpandemic era, peer support has switched to online support [3, 10]. This is natural since the use of the internet and social media is a part of daily life among young adults [11] and widely provides parenting-related information and social support [12].

Peer support involves two distinct perspectives: that of the individual receiving support and that of the peer providing it. It is known that acting as a peer supporter may give a supporter a sense of confidence and purpose in face-to-face support [13], however, peer supporters’ experiences of online support are currently unknown. From the perspective of breastfeeding mothers, online-provided peer support offers information, advice, emotional support, reassurance, and a sense of shared experience [7].

In addition to the internet and social media, mobile applications (software programs developed specifically for use on small, wireless computing devices) have for years been recognized as sources of breastfeeding information and support [14]. A review conducted in 2018 identified 82 breastfeeding-related apps [15]. However, studies regarding online breastfeeding peer support have focused mainly on social media and other forms of internet-based support [3, 7, 10] leaving peer support applications in shadows.

Apart from peer support, some studies have focused on application-based breastfeeding information and professional support. Some of those have links to breast milk substitute companies [16] and for-profit organizations [17, 18], raising questions of their trustworthiness and ethics. When discussing online breastfeeding interventions in general, it is important to carefully evaluate their compliance with the International Code of Marketing of Breast-milk Substitutes [19]. However, some breastfeeding-related nonprofit applications have shown good quality [20], usability [21], usefulness [21], acceptability [20] and feasibility [22] among breastfeeding mothers, although some technical difficulties have emerged, which negatively impacts the user experience [20, 21].

For modern applications, functionality (i.e., the application works without errors) is important but not sufficient for the users. The applications should be enjoyable, easy to use, and enhance user satisfaction and engagement [23]. These aspects, namely feasibility [24], can be measured in terms of appropriateness [25, 26], acceptability [25, 26], and usability [27, 28]. Appropriateness refers to the degree of the perceived fit and relevance for the current setting and how well the intervention is able to address the particular issue [25]. Acceptability, on the other hand, describes the levels of agreement, palatability, and satisfactoriness [25], whereas usability refers to how easy and efficient the use of the application is [27, 28]. Together, these three aspects of feasibility are interrelated [25, 26].

Breastfeeding is widely recognized to require both professional and peer support. Online peer support offers mothers accessible information, practical knowledge, skill development, and a sense of community. Mobile applications represent a relatively new form of delivering such support in digital environments. Although these applications are emerging as promising tools for peer-based breastfeeding support, their feasibility remains unexplored. Without evidence of feasibility, the potential benefits of app-based peer support cannot be fully realized.

## Methods

### Aim

The aim of this study was to examine the feasibility of Breastfeeding Peer Support Application (the App) from the viewpoints of peer supporters and breastfeeding mothers.

### Design and setting

This is a feasibility study with both qualitative and quantitative data, combining the perspectives of volunteer peer supporters and breastfeeding mothers. The study was conducted in Finland, which is a part of the Nordic countries characterized by universal health care and a comprehensive social support system, including long maternity and family leaves. Free public maternity and child health care are provided for all pregnant mothers and families. Breastfeeding counseling and support are part of maternity and child health care services [29]. In Finland, 94% of mothers initiate breastfeeding, and 50% of infants are exclusively breasfed at four months of age [30].

Trained peer support for breastfeeding is offered by the Finnish Association for Breastfeeding Support, a third-sector organization. For peer supporters, the association provides comprehensive education (based on WHO 20-hour breastfeeding counseling education and information of peer support), continuous support from coordinators (employers of the association) and peer support in supporters’ own virtual group. The association provides families with comprehensive, evidence-based online resources on breastfeeding and face-to-face and online support to more than 20,000 parents each year and delivers peer support through trained peer supporters via social media, webinars, support group meetings, and, most recently, a mobile application.

The mobile application, developed independently of this study to provide diverse forms of support for breastfeeding mothers, is called Breastfeeding companion (Mesensei), and it aims to provide one-to-one breastfeeding peer support for breastfeeding mothers. The App is available for both IoS and Android, and the use is free of charge. Support-seeking mothers are able to see peer supporters’ anonymous usernames and expertise areas and search for specific themes and issues (e.g., toddlers, biting). The peer supporters have three codes on the basis of their availability: green (promise to answer within 24 h), gray (promise to answer within 2–3 days), and hidden (not available). Mothers use nicknames as well and therefore remain anonymous. Mothers start the conversation by sending a message to one selected peer supporter. Conversations are saved, and mothers can continue conversation later and gain support from one peer supporter even for several months.

### Participants and procedures

The qualitative data were collected from breastfeeding peer supporters, who served in the App. The invitation to participate was sent via email by the associations’ employees, using peer supporters’ mailing list. There were approximately 100 peer supporters in the App, but not all actively serving as a peer supporter, so it is not possible to determine the exact population size. Potential participants were instructed to contact the researcher via email. In response to the email, an information sheet describing the study’s aim, procedure, voluntary nature and handling procedures of private data was sent. After consenting to participate, the participants filled out an electronic form (REDCap electronic data capture tool) [31, 32] including informed consent to participate and questions regarding background information.

The quantitative data from breastfeeding mothers were collected via the App from June to September 2022. The link to the questionnaire (REDCap) was embedded in the App, with a short invitation to participate. All breastfeeding mothers who had experience with the App were invited without specific inclusion criteria. The exact sample size could not be determined, as the app was freely downloadable and the number of mothers using it could not be tracked. The information sheet was available at the beginning of the questionnaire. Filling the survey was sought to express a consent to participate. No identifiable information was collected.

### Qualitative data collection: focus group interviews with peer supporters

In total, three focus group interviews were conducted in June and August 2022 via Zoom. The participants were asked to describe (1) what motivated them to start serving as peer supporters in the App, including participants’ preliminary thoughts of pros, cons and concerns; (2) their experiences of being peer supporters in the App, including benefits, issues, and differences related to other forms of peer support (e.g., face-to-face support); and (3) potential development and improvement ideas. The duration of the interviews varied from 66 min to 73 min (208 min in total). The interviews were transcribed verbatim, resulting in 26,600 words of written text.

### Quantitative data collection: feasibility survey of breastfeeding mothers

The survey included a background information questionnaire, two validated questionnaires, and one non-validated questionnaire related to feasibility. The intervention appropriateness measure (IAM) and acceptability of intervention measure (AIM) are both 4-item measures with 5-point Likert scales ranging from completely disagree to completely agree [26]. Both questionnaires were developed to evaluate the success of implementation processes. Appropriateness refers to the perceived fit from a technical or social perspective, whereas acceptability reflects the perception that the provided service is agreeable or satisfactory [26]. Usability was measured via items related to learnability, efficiency, memorability, errorlessness and satisfaction [27]. These items were operationalized for this study by the researchers and measured with a 5-point Likert scale from completely disagree to completely agree. In addition, open-ended questions concerning possible problems in the App, solutions to the problem and open feedback were included at the end of the survey.

### Qualitative data analysis

Peer supporters’ experiences were analyzed via inductive content analysis [33]. The transcribed data were coded on the basis of the research aim from the perspective of peer supporters. The codes (*n* = 646) were organized into subcategories (*n* = 28) on the basis of their internal homogeneity and external heterogeneity. The analysis proceeded to form categories (*n* = 4). The analysis was conducted via Atlas.ti for Windows (Scientific Software Development GmbH, version 25.0.1). Both researchers independently contributed to the analysis. The first author conducted the initial coding and developed the preliminary categories, while the last author confirmed the codes and further refined the sub-categories and categories. Throughout the process, regular discussions were held to ensure transparency and enhance the trustworthiness of the qualitative analysis. Background information was analyzed via descriptive statistics.

### Quantitative data analysis

The background information and views of the breastfeeding mothers were analyzed via descriptive statistics. In this study, the scales demonstrated good internal consistency (Cronbach’s alpha of 0.90 for IAM and 0.94 for AIM). The newly developed usability scale demonstrated good internal consistency (Cronbach’s alpha = 0.94). The scales showed strong (Spearman’s) correlations between IAM and AIM (*r* = .80, *p* < .001), IAM and usability (*r* = .65, *p* < .001), and AIM and usability (*r* = .68, *p* < .001), suggesting the concurrent validity of the scales.

The associations between background information and IAM, AIM, and usability were studied via Spearman’s rank-order correlation and the Mann‒Whitney U test. Nonparametric tests were used because of the nonparametric nature of the variables and the relatively small sample size. A subgroup analysis of mothers with unresolved breastfeeding problems was conducted using the non-parametric Mann-Whitney U test and Fisher’s exact test. SPSS Statistics, version 29.0 (IBM), was used for the analysis. The responses to the open-ended questions were categorized.

### Ethics

Ethical approval was obtained from the Ethics Committee for Human Sciences at the University of Turku (Statement 9/2022). In addition, permission to conduct the study was granted by the Finnish Association for Breastfeeding Support. Participants were recruited in collaboration with the Association, using the association’s employee as the contact person. Written informed consent was obtained from each peer supporter prior to the focus group interview. Separate informed consent was not requested from the breastfeeding mothers; completion of the survey was considered to indicate consent. The study was conducted in accordance with the principles of good scientific practice.

## Results

### Characteristics of peer supporters

Eight peer supporters participated in the focus group interviews. The median age of the peer supporters was 32.5 years (range 22–39 years), and each of them had at least two children (range 2–4). Five of the peer supporters had university-level education, and three of them were nurses; thus, some breastfeeding education was included in their studies. The participants had at least one year of experience being a breastfeeding peer supporter, and three of them had been peer supporters for more than four years. All participants had completed the peer support mother course by the Finnish Association for Breastfeeding Support before they started to practice as peer supporters. With respect to the app, some of the participants mentioned that they had received short orientation to use it.

### Feasibility from the viewpoint of peer supporters

The qualitative analysis resulted in four main categories: (1) Controlling the burden of peer support, (2) Balancing between sense of duty and the features of the App, (3) Disappearing versus rewarding contacts, and (4) Real-time support via the App is the present. A total of 14 subcategories were included in the main categories (Table 1).

**Table 1:** The main categories and subcategories describing feasibility from the viewpoint of peer supporters

Controlling the burden of peer support	Balancing between the sense of duty and the features of the App	Disappearing versus rewarding contacts	Real-time support via the App is the present
• Limiting the amount of peer support • Limiting the length of peer support contact • Independent of time and place • Personal format of peer support	• Need for peer support increased • Contacts in the App not divided evenly • Breastfeeding mothers enabled to choose a suitable peer supporter	• Breastfeeding mothers just disappear • Positive feedback motivates • Peer supporter shopping • Rewarding long-term support contacts	• Easy to use real-time support • Providing answers at own pace • Technical challenges and wishes for new features

### Controlling the burden of peer support

The App was considered a convenient way to control how much peer support work was included in peer supporters’ daily lives. Peer supporters said that limiting the amount of peer support in the App has been easier than they expected. However, it has not always been easy, and the resources for peer support are individual. In the app, the peer supporter can change her status from green to gray or hidden to be shown as unavailable. Peer supporters described regulating their availability according to the number of contacts.

All in all, when it’s voluntary, you also have to be able to limit yourself. Because it’s on the phone, it’s easy to keep it open a lot… But you just have to know yourself and know your own limits so that you don’t get burdened by something like this. (Interview #2)

Although the number of peer support contacts could be limited, peer supporters have struggled with how to limit the length of a single peer support contact. Beforehand, the peer supporters worried how they could end the contact if the breastfeeding problem was too challenging or outside peer support.

And at what point do you just have to steer forward. And sometimes in a very strict tone. That now I have given what I can give here. And the next address is this and that. That I can’t do anything else. (Interview #2)

One of the main reasons for joining the App was that it was independent of time and place. Moreover, being anonymous and identified as a peer supporter was seen as an important feature. Peer supporters valued the possibility of providing peer support according to their individual schedule and not disturbing their family time. They can, for example, conduct peer support while their own children are sleeping or while they are waiting for their children to be involved in hobbies.

To be able to do more of the support work, as it were, but then within your own schedules. (Interview #3)

Peer support via the App was more personal than was peer support via social media platforms. The interaction between the peer supporter and breastfeeding mother was evaluated as genuine, as the discussion was always private between two people.

### Balancing between the sense of duty and the features of the App

Peer supporters were dedicated to their support work, and they had a strong sense of duty. They felt that the need for peer support has increased, and the App responded well to that demand. The experienced benefits of the App had increased the willingness to use it. The App was meaningful for both peer supporters and breastfeeding mothers. Peer supporters considered the quality of support to be better because the support contact was intensive and dyadic. Peer supporters also had a separate closed group where they could consult each other to provide the best possible support for the mothers.

Peer supporters described struggling with the desire to help and their own resources. The participants described that the contacts by breastfeeding mothers had not been divided evenly among available peer supporters. Paradoxically, active peer supporters with green status rose to the top of the list of peer supporters and, by implication, were having increasingly more support requests. They wished that the App could automatically match even the number of contacts with each peer supporter. The peer supporters also wished for the possibility of transferring the support request to another peer supporter in the App if they did not have enough time or skills to solve the problem.

That in a way, that you’re thinking, well, I’m having some time right now, so I’ll put myself online that I’m green. And then you have ten contacts. You’re like I could talk to a couple of them and then suddenly you have to try to get them all answered until late at night. (Interview #2)

And of course, now that when there are no messages at all, that when you would have time to answer, there won’t be. So [the number of messages] varies. (Interview #1)

Peer supporters appreciated the feature that the breastfeeding mother can choose a suitable peer supporter on the basis of the expertise areas marked in the App via hashtags. Breastfeeding mothers are able to use hashtags as key words to find a suitable peer supporter for their problems. The possibility of choosing, however, caused some irrational fears of “*if no one wants me to be her peer supporter*” (Interview #3). Expertized areas were marked by the peer supporters themselves on the basis of their education and personal experience. The use of expertise areas brought confidence and fluency to support work, as the peer supporters felt that providing support was easy if the breastfeeding problem was considered an area that was familiar to them.

I’ve experienced that many times support seekers use hashtags [keywords indicating expertise areas] and reading those introductory texts. That gives you the confidence to go and support that person, because you know that at least that thing is familiar and safe to you, that you can know enough about it to be able to help directly without a massive search operation. So that also brings that ease to the support work. (Interview #2)

### Disappearing versus rewarding contacts

Regardless of mainly positive experiences of providing support via the App, the support contacts varied considerably. Some of the contacts were short and quick, and the problem was solved easily with one response; however, some of the contacts were challenging and complicated and demanded considerable background information.

Peer supporters said that some mothers just disappeared after the response was provided, which caused frustration and insecurity. The peer supporters were hesitant whether they helped or did not help the mother solve her breastfeeding problem. Some assumed that the problem was solved, but some peer supporters also thought of different options. They even thought if they provided such bad advice that the mother approached another peer supporter. Sometimes peer supporters considered sending a new message to ask how things are going with the mother. In contrast, some mothers thanked and provided many compliments after successfully solving their breastfeeding problems. Spontaneous and direct thanks and positive feedback motivated peer supporters to continue as they concretely understood that their support was meaningful. Sometimes positive feedback was provided for a long time after the initial contact and discussion between the mother and the peer supporter.

But then sometimes you are left wondering if the problem was solved or not. Or did a mother change to someone else, whether I performed somehow poorly. (Interview #1)

The mother tell you, for example, that the breast refusal has eased now and that now breastfeeding continues and feels good and that we got over that difficult phase, then it feels nice and rewarding. (Interview #3)

“Peer supporter shopping” is a phenomenon in which a support-seeking mother sends messages to several peer supporters at the same time. The mother might be in a hurry or stressful situation and wish for a quick response from anyone. However, the peer supporters also considered whether these mothers expected different answers and wanted to choose the best answers. All the peer supporters had not suffered from this “shopping”, but some of them experienced it frustrating and especially time-consuming. Peer supporters also thought that some support-seeking mothers had unrealistic expectations from the App, or, generally, from peer support. The mothers might have expected that the peer supporters would answer them immediately and give them some kind of magic trick to solve their problems all at once. Some mothers were not prepared to work themselves to achieve the desired result with their breastfeeding.

So in situations like this, even if there have been a couple of shoppers like this, who are looking for messages from many people, I think about how much energy they have taken away from me […] then I could have used that energy to support another person (Interview #1)

The App enabled long-term support contacts, which were rewarding from the perspective of peer supporters. The discussions were saved in the App, and it was easy to check what they had previously discussed if the mother contacted the same peer supporter again. Problem solving was more straightforward when background information and the starting point were already clarified. Peer supporters considered that they had succeeded in building trust if the mother returned to them later.

And then I’ve had a few that are like… For several months, she has always sent me a message with new problems and I have been able to go along, so to speak, so it has been a really nice thing. (Interview #3)

### Real-time support via the App is the present

Peer supporters had mixed feelings before the use of the App. Some of them thought that the App would be difficult to use, but these prejudices turned out to be wrong. Some others had positive expectations only. All the peer supporters considered the App beneficial, and it proved to be very easy to use. Peer supporters believe that some people are truly worried about issues related to baby care and breastfeeding and that they expect answers as soon as possible; thus, real-time breastfeeding support is needed. Sometimes help is crucial, and waiting even for a day or two days might negatively impact breastfeeding. The App enables requests and provides support anytime. Moreover, peer supporters suggested that in the future, an online emergency line in the App could provide the answer immediately.

And then there could be one more option — like, you’d show that you’re available right now. Kind of like an online status in a chat: ‘Now I’m focused on this,’ or ‘Now I’ve got time for this’. (Interview #2)

Regardless of the hectic nature of people’s lives today, the App enabled the peer supporters to answer and discuss at their own pace. Complicated problems required some processing, and the peer supporters wanted to carefully think and plan their response to be able to help the mother comprehensively. They also wanted to search for more information before sending their response. On the negative side, the peer supporters mentioned the challenges in written answers. The reader of a written text may sometimes interpret the message differently than the writer has meant.

The participants emphasized some technical challenges, such as problems with the App notifications. They found it truly negative that they could not rely on the notifications sent by the App. Peer supporters described that many contacts were delayed because no notifications appeared and they did not remember to visit the App to check new messages or contacts. Sometimes it was frustrating because they would have had time to discuss in the App but they were not aware of mothers’ requests. Another problem was the disappearance of the unfinished and unsaved message; however, it was fortunately not very common.

Only the lack of notifications has sometimes made it so difficult that I’m constantly afraid of not answering a message from there […] So it’s really sad that if someone goes unnoticed, that you must observe a bit. (Interview #2)

Peer supporters’ wishes for the new technical features of the App considered possibilities to attach videos in the conversations and phone calls or voice messages from peer supporters to the mothers. Statistics about the provided peer support would also be beneficial from the point of view of the peer supporters. The App could help limit the number of support contacts by changing the status from green to gray when the maximum number of contacts was reached. Furthermore, the peer supporters described many minor technical challenges but also considered them normal in the case of a new application. Most of these minor challenges did not impact feasibility or conversations with breastfeeding mothers.

In general, the peer supporters who participated in this study reported that the App responded well to the support needs of today’s mothers. People are accustomed to communicating with short messages; thus, it is also considered a suitable way to support breastfeeding.

And it’s a modern-day tool, no doubt. In general, when people like instant messaging on their device, in that sense. (Interview #2)

### Characteristics of breastfeeding mothers

A total of 81 respondents participated in the usability survey between June 2022 and June 2023. The median age of the participants was 31 years, ranging from 18 to 41 years. The participants were highly educated; 70% (*n* = 57) had polytechnic- or university-level education. The majority (*n* = 56, 69%) of the participants were breastfeeding their first-born infants. All but one participant reported the age of the baby they were breastfeeding. The median age of the breastfed infants was 5 months, ranging from 0 to 24 months.

### Feasibility from the viewpoint of breastfeeding mothers

The feasibility of the App was found to be very good. The measures showed high acceptability (AIM), appropriateness (IAM) and usability (Table 2).

**Table 2:** Feasibility of the App

Feasibility	Median (Q_1_-Q_3_)
Acceptability of Intervention Measure (AIM)	5.0 (4.3-5.0)
Intervention Appropriateness Measure (IAM)	4.8 (4.0–5.0)
Usability	4.5 (3.9-5.0)

Mothers’ age was not correlated with acceptability (*r* = .1, *p* = .48), appropriateness (*r* = .2, *p* = .14), or usability (*r* = .2, *p* = .10) of the App. Furthermore, the number of children (one vs. two or more) or mothers’ education (second grade vs. polytechnic or university level) was not associated with acceptability (*p* = .15 and *p* = .41), appropriateness (*p* = .84 and *p* = .51) or usability (*p* = .47 and *p* = .28), respectively.

Most of the respondents (*n* = 65, 80%) found a solution to their existing breastfeeding problem via the App. However, 15% (*n* = 12) of the participants reported that they had not found a solution, and four respondents did not provide any response. Mothers whose breastfeeding problem was resolved did not differ from those whose problem remained unresolved in terms of age (*p* = .56), educational background (*p* = .73), or parity (primiparous vs. multiparous; *p* = 1.000). Furthermore, no associations were found with perceived acceptability (*p* = .94), appropriateness (*p* = .87), or usability (*p* = .13). These findings suggest that the unresolved breastfeeding problems were not due to the usability of the App, but rather related to the nature or severity of the breastfeeding issue itself.

Altogether, 19 participants reported experiencing problems while using the app. The majority of these issues (*n* = 13) were related to sending or receiving messages. Eight participants reported that notifications for new messages either did not work or were significantly delayed. In five cases, the messages failed to reach the recipient, resulting in delayed support or responses. Moreover, the participants reported difficulties with calls (*n* = 2), trouble understanding the symbols in the App (*n* = 1), and being unable to attach videos to the App (*n* = 1). Additionally, two participants stated that the App did not work properly, but they did not provide further details.

General feedback was provided by 27 participants, and it was mostly positive. Sixteen participants described the App as magnificent, and the support provided through it was invaluable. The App was praised for being low-threshold and easily accessible. Many participants also noted that support was often provided on the same day their question or request was sent. One participant commented, *“Works well and has been a lot of help. Owing to this app*,* breastfeeding continued until the baby was 1.5 years old. (ID 18)*

Critical feedback was also provided, along with some constructive development ideas (*n* = 11). Four participants reported difficulty in choosing a peer supporter. It was not always clear whether the selected peer supporter was available or if she was the right person to address the specific breastfeeding issue. Six pieces of feedback were directed at the App itself. Some participants noted that certain app functions seemed to conflict with each other and that notifications for messages were not working consistently. One participant suggested adding more detailed videos demonstrating breastfeeding positions and proper latch techniques.

The idea of the App is very good. Occasionally, the App does not work as it should and that impacts the user experience. (ID 36)

The App should be further developed and re-evaluated, for example, the words used. (ID 46)

## Discussion

Breastfeeding Peer Support Application seemed to be a feasible tool for providing breastfeeding support from the perspectives of both peer supporters and breastfeeding mothers. In qualitative focus groups, peer supporters appreciate the possibility of controlling the amount of peer support work and the ability to balance it with their own resources. Peer supporters had a strong sense of duty, and some of the features of the App helped them avoid overusing their resources; however, some features still need improvement. The peer support contacts with breastfeeding mothers were mostly rewarding, but a few suddenly breaking and disappearing contacts caused frustration and insecurity. The App was found to be present regardless of certain technical challenges faced. On the basis of the survey results, the end-users, breastfeeding mothers, were satisfied with the App; it was ranked high in acceptability appropriateness and usability regardless of mothers’ age, education level or number of children. Like peer supporters, breastfeeding mothers reported some technical problems. In terms of feasibility, the findings of this study were in line with other studies demonstrating a good feasibility of peer support applications [34, 35], although opposite findings have also been presented [36].

Since peer supporters are volunteers who engage in peer support during their free time, the ability to manage their workload through the App was highly appreciated. Practical constraints and personal resources—such as the need to provide support at specific times and locations—pose challenges for peer supporters [13]. Peer supporters providing face-to-face support are very committed to and passionate about their role [37]. Similarly, in this study, a strong sense of duty and a desire to help breastfeeding mothers had previously limited peer supporters’ ability to manage the demands of their role. However, the App’s functionalities help mitigate this burden by offering greater control. When peer support on social media platforms (e.g., Facebook) was compared with that on mobile applications, the App was rated more favorably because of its unique features. Peer supporters could set up their availability, and the support interactions remained private, free from external interference or false comments [7], ensuring a confidential space for conversations between mothers and peer supporters.

The need for breastfeeding peer support was recognized by both peer supporters and breastfeeding mothers. The App effectively responded to this growing demand, as it is easily accessible and constantly available [3]. Most mothers reported receiving support and solutions to their challenges. It is well established that breastfeeding mothers often value the emotional and practical assistance provided by peer supporters [4, 13] and that peer support is responsive to the needs of breastfeeding mothers [7]. Based on this study, peer supporters also found that support interactions were rewarding, particularly when they were able to help mothers. At best, the breastfeeding peer support application served both groups well, enabling a satisfying experience for all users [37].

Mutual respect and trust are essential elements of breastfeeding peer support [38]. Peer supporters need to understand the unique circumstances of each breastfeeding mother [37]. However, some peer supporters felt that their efforts were occasionally misused through “shopping for peer supporters”—that is, when mothers asked the same question to multiple supporters simultaneously. This phenomenon caused frustration, as it made some peer supporters feel that their work was not fully respected. On the other hand, it is possible that the mother was facing an acute issue and was simply trying to find a solution by any means necessary. Peer supporters suggested establishing an acute support line for the App. However, adding “emergency peer support” is not a solution to the increasing need for breastfeeding support. The Finnish Association for Breastfeeding Support [39] reported having more than 21 000 support requests in 2024. The number represents approximately half of the infants born yearly in Finland. The increasing number of support requests reflects challenges in breastfeeding support in the health care system. Families cannot reach health care professionals outside office hours; thus, peer support is the only possibility for distressed parents.

Technical issues, such as missing notifications or difficulties in sending or receiving messages, were reported by participants in both groups. While such problems are not uncommon with digital devices and applications, they can be particularly critical in this type of support format. If messages fail to reach either peer supporters or breastfeeding mothers, the continuity of peer support is disrupted, potentially affecting breastfeeding outcomes. Moreover, trained volunteer peer supporters deserve access to a reliable and user-friendly system, so their time can focus on providing breastfeeding support rather than troubleshooting technical issues.

Although this study suggests that app-based breastfeeding peer support is generally feasible, there is still a lack of evidence regarding its effectiveness in extending breastfeeding duration. This gap in evidence applies broadly to all forms of online breastfeeding peer support [3]. Moreover, as this study was conducted in a high-income country, the results are likely not generalizable to middle- and low-income countries. Therefore, country- and culture-specific studies are still needed. In the future, if app-based breastfeeding peer support demonstrates both high feasibility and effectiveness, it should be integrated into formal healthcare systems as a complementary form of support.

### Strengths and limitations

A key strength of this study is the inclusion of perspectives from both peer supporters and breastfeeding mothers, providing a comprehensive understanding of the App’s feasibility. Additionally, the use of multiple methodological approaches enhances the robustness of the study [40]. Qualitative analysis was conducted by two independent researchers, and direct quotes from all participant groups were included to increase the credibility of the findings. Dependability was supported by the relatively short data collection period and close collaboration among experienced researchers. Transferability of the findings is limited by the small sample size and variations in breastfeeding-related norms and support practices across different countries and regions [41]. However, the global need for breastfeeding support suggests that a smart device application could be feasible in various settings.

With respect to the quantitative survey, the small sample size, the relatively homogeneous sample with a high educational background, and the absence of a power analysis limit the generalizability of the findings. User engagement statistics were not collected, although they would have been useful in describing the utility of the App. While part of the instrument set (IAM, AIM) had been previously validated, additional non-validated questions were also used to assess feasibility. No official pre-testing was carried out for the non-validated questions included in the study. Nevertheless, all the instruments demonstrated good reliability in this study [42].

Both researchers have professional backgrounds in healthcare, promoting breastfeeding as a NICU nurse and a midwife, respectively. In addition, both have previously conducted breastfeeding-related research. New approaches to promoting breastfeeding are of interest to both researchers, and while their breastfeeding-positive mindset may have influenced the qualitative analysis, both are experienced in conducting research.

## Conclusions

The breastfeeding peer support application demonstrated good feasibility from the perspectives of both trained peer supporters and breastfeeding mothers. The App was easily integrated into the daily lives of both user groups. Peer supporters appreciated the opportunity to provide support within their available resources, and most mothers found effective solutions to their breastfeeding challenges. While some user behavior issues and technical problems were reported, numerous suggestions for improvement were also provided. Overall, theApp was regarded as a highly promising tool for supporting breastfeeding. This study offers valuable insights into key considerations for developing and implementing peer support applications. Such apps should be engaging while also offering users the ability to control and limit their usage. In the future, the effectiveness of these applications should be evaluated through controlled trials focusing on long-term breastfeeding outcomes. Furthermore, effective implementation strategies for these interventions should be developed.

## Data Availability

The data are not publicly available due to their sensitive nature but are available from the corresponding author upon reasonable request.

## Abbreviations

AIM: Acceptability of intervention measure
App: Application
IAM: Intervention appropriateness measure
WHO: World Health Organization
